# Bioresponsive matrices in drug delivery

**DOI:** 10.1186/1754-1611-4-15

**Published:** 2010-11-29

**Authors:** Jin-Oh You, Dariela Almeda, George JC Ye, Debra T Auguste

**Affiliations:** 1 School of Engineering and Applied Sciences, Harvard University, Cambridge, MA 02138, USA

## Abstract

For years, the field of drug delivery has focused on (1) controlling the release of a therapeutic and (2) targeting the therapeutic to a specific cell type. These research endeavors have concentrated mainly on the development of new degradable polymers and molecule-labeled drug delivery vehicles. Recent interest in biomaterials that respond to their environment have opened new methods to trigger the release of drugs and localize the therapeutic within a particular site. These novel biomaterials, usually termed "smart" or "intelligent", are able to deliver a therapeutic agent based on either environmental cues or a remote stimulus. Stimuli-responsive materials could potentially elicit a therapeutically effective dose without adverse side effects. Polymers responding to different stimuli, such as pH, light, temperature, ultrasound, magnetism, or biomolecules have been investigated as potential drug delivery vehicles. This review describes the most recent advances in "smart" drug delivery systems that respond to one or multiple stimuli.

## Introduction

Polymeric materials that respond to a stimulus are often called "smart" or "intelligent" due to their intrinsic ability to alter their physical or chemical properties. For the majority of the polymers that fall into this category, the response to a change in the surrounding environment is not only quick, on the order of minutes [1,2] to hours [3,4], but also reversible, mimicking the dynamics observed in natural polymers, such as proteins, polysaccharides, and nucleic acids in living organic systems [5]. The response to stimuli is manifested in many forms: individual chain dimension/size, shape, surface characteristics, secondary structure, solubility, and degree of intermolecular association. These unique capabilities have been applied to a diverse range of applications, including: drug delivery [4,6-8], diagnostics [9,10], biological coating technologies [11,12], biosensing [10,13], and microfluidics [14].

Conventional drug delivery methods physically entrap molecules within a polymer lattice; drug is released slowly by diffusion or upon degradation of the network. These methods typically result in an early peak in plasma drug concentration followed by a steady, linear release. This is far from ideal because the local drug concentration and location of delivery is not precisely controlled. Below the therapeutic dose, the drug is ineffective whereas high concentrations of drug may be toxic or lead to undesirable side effects. Polymers have been used to tailor drug release, which maintains the drug concentration within the desired therapeutic range. However, such controlled release systems are insensitive to metabolic changes in the body and are unable to modulate drug release nor target the drug to diseased tissue. This lack of control has motivated the exploitation of bioresponsive polymers as drug carriers.

As early as the 1950 s, stimuli responsive hydrogels have been studied for drug release [15]. Since then, polymers that react to different stimuli have been developed. These stimuli include pH [16-20], ionic strength [21], and the presence of metabolic chemicals (e.g., enzymes or antigens) [22,23]. Such stimuli may enable a drug carrier to distinguish between diseased and healthy tissue. More recently, drug carriers that respond to magnetic fields [24], light [25], radiation [26], and ultrasound [27] have also been developed. These external stimuli allow for greater control over when and where the drug is released. By tuning the formulation or chemical moieties of the polymer, the sensitivity to the stimuli can be precisely controlled. This review aims to provide an overview of how responsive polymers may be used to improve drug delivery.

## Stimuli-responsive materials for drug delivery

### pH-sensitive drug delivery

pH-sensitive polymers (see Table 1) have garnered much attention in the fields of drug delivery [28,29], gene delivery [30], and insulin delivery [31]. Generally, pH-sensitive polymers have weak acids or bases with *p*K_a _values between 3 and 10 [32]. Carboxylic, sulfonate, and primary or tertiary amino groups exhibit a change in ionization state as a function of pH. Transitions in solubility, conformation, and swelling arise due to changes in ionization, where specific polymer groups switch between a neutral and charged state (e.g., poly(N,N-dimethylaminoethyl methacrylate (DMAEMA) [33-35]) or a hydrophilic and hydrophobic state (e.g., poly(*N*-iso-propylacrylamide) (PNIPAm) [36-38]).

**Table 1:** Stimuli-sensitive drug delivery.

Stimulus	Carrier type	Payload	Reference
pH	PPADK	Dexamethasone	[45]
	
	DMAEMA/HEMA	Paclitaxel	[3]
	
	PC/DAP liposomes	siRNA	[51]

Temperature	PNIPAm/PLGA	Paclitaxel	[36]
	
	MPPC/DPPC/HSPC/DSPEC-PEG-2000	Doxorubicin	[54]
	
	FA/PDMA/PNIPAm	Dipyridamole	[57]

Light	Cu chlorophyllin/PNIPAm	None reported	[60]
	
	Quinone-methide	Nile Red	[63]
	
	Au/meso porous silica	Paclitaxel	[61]

Ultrasound	Pluronic P105/N,N-diethylacrylamide	Doxorubicin	[66]
	
	DPPC:DPPE-PEG2000 liposomes	Calcein	[67]

Glucose	poly(methacrylic acid-g-ethylene glycol) with glucose oxidase, PNIPAm with phenylboronic acid	Insulin	[69-72]
	
	PNIPAm or carboxymethyl dextran with con. A	None reported	[73,74]

Enzyme	PEG diacrylate/human neutrophil elastase-sensitive peptide	None reported	[75]
	
	Fibrin/β-nerve growth factor fusion proteins	β-nerve growth factor	[76]
	
	sPLA_2_-degradable retinoid lipid pro-drug	Retinoid lipid pro-drug	[77]

Magnetic	Magnetite	Squalenoyl gemcitabine (SQdFdC)	[78]
	
	Poly[aniline-*co*-*N*-(1-one-butyric acid)] aniline (SPAnH)/iron oxide	Epirubicin	[79]
	
	Polylactide/nanocrystalline magnetite	Paclitaxel	[80]
	
	Egg-PC/maghemite/PAH/PSS	Calcein	[81]

Eisenberg et al. investigated pH-sensitive polymer swelling to control the release of drug molecules [15]. Since then, several biocompatible and biodegradable pH-sensitive polymers have been developed. Unfortunately, few pH-sensitive polymers have been used for drug delivery systems because of their limited sensitivity near the pH of blood (pH 7.4). For example, natural polymers (alginate [39,40], chitosan [41,42], and carrageenan [43]) and synthesized polymers (poly(acrylic acid) (AA) [44] and poly(methacrylic acid) (MAA) [45]) exhibit a high swelling property at high pH due to ionizable functional groups on the polymer backbone or side chain. These polymers are not responsive under most physiological conditions, albeit the gastrointestinal system.

**Figure 1:**
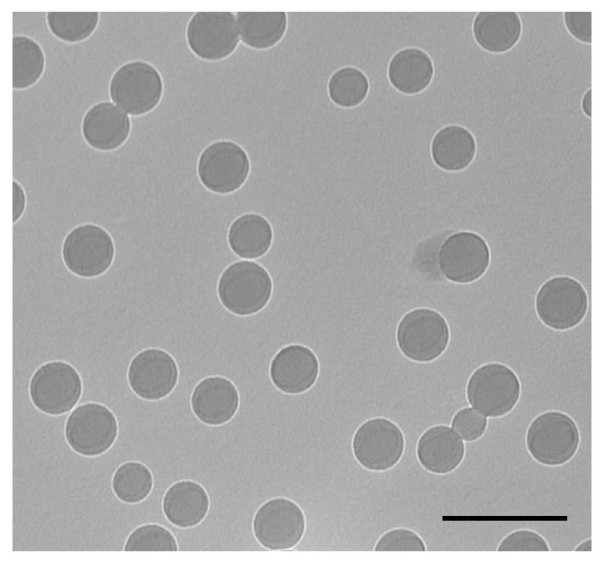
**Transmission electron microscope image of DMAEMA/HEMA nanoparticles used for drug delivery**. Scale bar is 500 nm [3].

**Figure 2:**
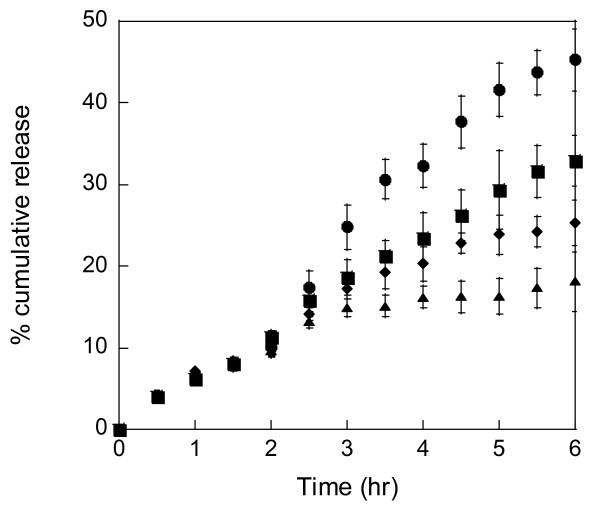
**Triggered paclitaxel release was observed by incubating 10/90 (mol/mol) DMAEMA/HEMA nanoparticles crosslinked with 3 mol% TEGDMA for 2 hours at pH 7.4 (black triangle) followed by a reduction in pH to either 6.8 (black circle), 7.0 (black square), or 7.2 (black diamond) for 4 hours**. The error is the standard deviation of the mean, where n = 3 [3].

**Figure 3:**
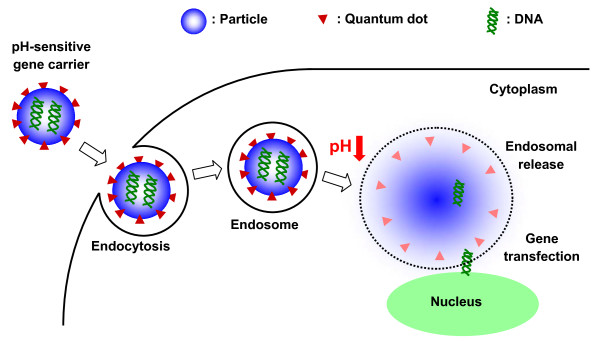
**Schematic illustration of the delivery of pH-sensitive gene carriers**. For example, the DMAEMA/HEMA nanoparticle releases DNA in the low pH endosome [49].

Systemic delivery requires that drug carriers respond to small changes in pH, near pH 7.4. In 2005, Heffernan and Murthy developed an acid-sensitive biodegradable drug delivery vehicle using poly(1,4-phenyleneacetone dimethylene ketal) (PPADK), which contains ketal linkages allowing for acid-catalyzed hydrolysis of the polymer into low molecular weight hydrophilic compounds. Thus, the release of drug molecules is accelerated under acidic conditions [46]. You and Auguste synthesized a series of pH-sensitive nanoparticles comprised of DMAEMA and 2-hydroxyethyl methacrylate (HEMA) (Figure 1) [3]. DMAEMA is a pH-responsive monomer that has a tertiary amine functional group with a *p*K_a _of 7.5 [47]. *In vitro *results support that the drug would remain within the particle during circulation; upon exposure to a low pH environment (e.g., a tumor [48]), the particle would swell resulting in release of the drug. Monodisperse, pH-sensitive DMAEMA/HEMA nanocarriers encapsulating paclitaxel exhibited pH-dependent release kinetics (Figure 2). The particles had a high volume swelling ratio at low pH, low crosslinking density, and high content of DMAEMA. A similar series of particles were used for gene delivery, where triggered release of plasmid DNA within the low pH endosome was optimized [49,50]. Plasmid DNA for green fluorescent protein was encapsulated. HeLa cells were successfully transfected with a dependence on the swelling ratio and crosslinking density (Figure 3).

pH-dependent liposomes have also been used to trigger the release of drug within acidic environments. Auguste et al. formulated liposomes with variable surface charge by varying the lipid composition [51]. pH-sensitive liposomes were composed of a zwitterionic lipid (phosphatidylcholine) and a titratable lipid (dimethylammonium propane) with a *p*K_a _of 6.7. This allowed the liposome's net charge to become cationic upon decreasing the pH. pH-dependent liposomes may be shielded from the immune system using poly(ethylene glycol) (PEG)-b-polycation polymers. The polycation block electrostatically anchored the PEG polymer to the liposome surface at pH 7.4, but released the polymer at pH 5.5 due to electrostatic repulsion between the cationic polymer and cationic liposome surface. The triggered release of adsorbed PEG-b-polycation polymers from pH-dependent liposomes may protect the drug carrier from immune recognition during circulation (pH 7.4) and allow subsequent intracellular delivery of siRNA within the endosome. The bare liposome maintains the membrane disruption/fusion capability [52,53].

### Temperature-sensitive drug delivery

Increases in temperature are associated with several disease states (e.g., cancer [54,55]). Thermo-responsive drug carriers have been employed to release their payload within environments above the physiological temperature. Thermo-sensitive polymers exhibit a phase transition in solution at a temperature known as the lower critical solution temperature (LCST). For example, PNIPAm, a well-studied thermo-responsive polymer, undergoes a reversible phase transition in aqueous solution from hydrophilic to hydrophobic at its LCST of approximately 32°C. Chemical modifications of PNIPAm have been effective in controlling the LCST [56]. In 2005, Liu et al. synthesized poly(*N*-isopropylacrylamide-*co*-N,N-dimethylacrylamide)-*b*-poly(D,L-lactide-*co*-glycolide) micelles for controlled paclitaxel delivery [36]. Paclitaxel release was accelerated when the physiological temperature was raised above the LCST. The paclitaxel-loaded micelles were more effective in killing human breast carcinoma cells at 39.5°C than 37°C. De and colleagues developed folate-conjugated, thermo-responsive block copolymer micelles. Folate is known to bind to several cancer cell types [57]. The drug release studies from folate-conjugated PNIPAm-DMA micelles demonstrated a temperature-responsive drug release. Delivery of paclitaxel at the tumor site can alter the overall drug biodistribution. Needham et al. developed temperature-sensitive liposomes containing doxorubicin [54]. Their liposome formulation, composed of 1-palmitoyl-2-hydroxy-*sn*-glycero-3-phosphocholine (MPPC), 1,2-dipalmitoyl-*sn*-glycero-3-phosphocholine (DPPC), hydrogenated soy *sn*-glycero-3-phosphocholine (HSPC), and 1,2-distearoyl-*sn*-glycero-3-phosphoethanolamine-*N*-polyethylene glycol 2000 (DSPE-PEG-2000), was optimized to rapidly release the drug under mild hyperthermic temperatures (39°C to 40°C). Changing the drug biodistribution can increase therapeutic efficacy.

### Light-sensitive drug delivery

Light (ultraviolet or visible) is a desirable external stimulus for drug delivery systems because it is inexpensive and easily controlled. Light-sensitive drug carriers are fabricated from polymers that contain photo-sensitizers such as azobenzene, stilbene, and triphenylmethane [37,58,59]. Suzuki and Tanaka have investigated visible light-responsive hydrogels using the trisodium salt of copper chlorophyllin in PNIPAm hydrogels [60]. When light is applied to the hydrogels, the chromophore absorbs the light, increasing the local temperature of the hydrogel. The resulting temperature change alters the swelling behavior. Vivero-Escoto et al. prepared gold-capped mesoporous silica nanospheres for photo-induced intracellular release of drugs in human cells [61]. The 100 nm silica nanospheres were capped with 5 nm gold nanospheres and functionalized with a cationic photo-reactive linker. Photoirradiation using ultraviolet (UV) light for 10 min at 0.49 mW/cm^2 ^cleaved the photolabile linker, causing uncapping of the silica due to charge repulsion between the gold and silica nanospheres, allowing drug to be released [61,62]. Fomina et al. developed a novel light-sensitive polymer containing a quinone-methide moiety [63]. Nile Red, a hydrophobic dye, was released from nanoparticles after only one minute of 350 nm light exposure. Light can be effective in modulating drug release because it can be used to increase the local temperature and to cleave bonds.

### Ultrasound-sensitive drug delivery

Ultrasound has been shown to trigger drug release by raising the local temperature or causing cavitation [64]. Both processes can increase the permeability of cell membranes and accelerate polymer degradation [65]. Ultrasound-sensitive vehicles have the potential to treat tumorigenic cancers due to their invasive character, ability to penetrate deeply into the human body, and ease of control. In 2002, Pruitt and Pitt investigated ultrasound-mediated doxorubicin release using stabilized Pluronic P105 micelles [66]. Doxorubicin was encapsulated within polymeric micelles composed of 10% Pluronic P105 and N,N-diethylacrylamide and delivered systemically to rats. Application of low-frequency ultrasound at the tumor site resulted in doxorubicin release; this resulted in a significant reduction in tumor volume. Lin et al. have investigated the physical and chemical properties of lipid membranes subjected to ultrasound treatment [67]. They showed that high permeability resulting from ultrasound treatment is correlated with lipid packing and can be useful for efficient drug release and ultrasound-mediated DNA transfection. In 2007, Ferrara et al. reviewed that small gas bubbles, used to enhance ultrasound contrast, can be used for drug delivery applications and monitoring [68]. When driven by an ultrasonic pulse, small gas bubbles oscillate with a wall velocity on the order of tens to hundreds of meters per second and can be deflected to a vessel wall or fragmented into particles on the order of nanometers. Also, a focused ultrasound beam can be used for disruption of delivery vesicles and blood vessel walls, which offer the opportunity to locally deliver a drug or gene. Ultrasound does not damage the surrounding tissue, making it attractive for triggering drug release.

### Biomolecule-responsive drug delivery

The presence of biomolecules specific to an organ or disease state may be useful to regulate the release of drugs. Biomolecules can either participate in a chemical reaction or result in cleavage of a chemical bond. In this section, we will discuss the use of hydrogels that are (1) responsive to glucose and (2) use enzymes to facilitate hydrogel degradation.

Glucose-responsive hydrogels have been investigated for self-regulating the release of insulin for the treatment of diabetes. Early studies have been largely based on the combination of glucose oxidase with polyelectrolyte hydrogels that exhibit pH-responsive swelling or shrinking behavior [69,70]. As glucose diffuses within the hydrogel, entrapped glucose oxidase catalyzes its conversion to gluconic acid, lowering the local pH of the gel and resulting in swelling and the subsequent release of insulin. However, the efficiency of glucose oxidase decreases with pH. PNIPAm coupled with phenylboronic acid has been investigated as a glucose-responsive system [71,72]. Introduction of phenylboronic acid decreases the volume phase transition temperature. The hydrogel swells in the presence of glucose. More recently, Miyata et al. demonstrated that biomolecular complexes such as the carbohydrate-binding lectin, concanavalin A (Con. A), could be coupled with PNIPAm to achieve reversible swelling or shrinking in response to stepwise changes in glucose concentration [73]. Zhang et al. also utilized Con. A as the cross-linker for carboxymethyl dextran (CM-dextran) based hydrogels. Competitive displacement between Con. A and terminal glucose moieties on dextran by free glucose changed both the morphology and permeability of the gel [74]. Although these systems triggered insulin release through volumetric changes, regulating the rate and reliability of release remains a challenge.

Researchers have also exploited the presence of site or disease specific enzymes in drug delivery by incorporating enzyme-cleavable peptides within hydrogels. For example, Aimetti et al. prepared a PEG hydrogel drug delivery system which incorporated human neutrophil elastase (HNE)-sensitive linkers for the treatment of inflammation. HNE is a serine protease secreted by neutrophils, which accumulates at sites of inflammation. HNE-sensitive peptides were synthesized using solid phase Fmoc chemistry and their degradation kinetics were characterized. The rate of substrate degradation can be tailored by the incorporation or substitution of specific amino acids. Local, controlled release from hydrogels containing HNE-sensitive peptides was achieved in the presence of HNE as visualized by fluorescence energy resonance transfer (FRET) [75].

Growth factor delivery, controlled by enzymes involved in tissue regeneration, has also been investigated. Sakiyama-Elbert et al. designed the delivery of beta-nerve growth factor (β-NGF) from fibrin matrices as a nerve regeneration therapy. They synthesized β-NGF fusion proteins with an enzymatically degradable linker that served as the covalent anchor to the fibrin matrix and thus prevented a potential loss of enzymatic activity [76]. Researchers have also exploited the enzyme-triggered degradability of certain prodrugs. Pedersen et al. investigated anti-cancer retinoid lipophilic drugs that are covalently attached to phospholipids. These prodrugs have a lipid backbone that is degradable by secretory phospholipase A_2 _(sPLA_2_) IIA. The prodrugs self-assembled into liposomes, which were susceptible to degradation by (sPLA_2_) IIA. *In vitro *studies utilizing MT-3 breast carcinoma and HT-29 colon adenocarcinoma cell lines demonstrated high cytotoxicity of prodrug liposomes only in the presence of the (sPLA_2_) IIA enzyme [77].

### Magnetic-sensitive drug delivery

Magnetic drug delivery systems possess three main advantages: (1) visualization of drug delivery vehicles, (2) ability to guide and control movement of drug carriers through magnetic fields, and (3) thermal heating which has been used to control drug release or produce tissue ablation. Magnetic drug carriers like magnetite, maghemite, cobalt ferrite, and carbonyl iron are biocompatible, non-toxic and non-immunogenic [78]. Arias et al. utilized magnetite to produce magnetic core/shell nanoparticles for drug delivery. The nanoparticles consisted of a magnetite core with a self-assembling squalenoyl gemcitabine, an amphiphilic anti-cancer drug, shell. Optical microscopy images showed the alignment of the core/shell nanoparticles under the influence of a 0.2 T magnetic field [78]. Liu et al. reported *in vitro *and *in vivo *studies of poly[aniline-co-*N*-(1-one-butyric acid)] aniline (SPAnH) nanoparticles encapsulating iron oxide (Fe_3_O_4_). To overcome the blood-brain barrier, the authors utilized focused ultrasound to temporarily disrupt the barrier and increase permeability. Their results showed that an estimated 0.16 ± 0.03 mM of magnetic nanoparticles were delivered to brain tumors in Sprague-Dawley rats; this was estimated to be 15-fold higher than the therapeutic range [79]. Magnetic nanoparticles have also been proposed as a component in drug eluting stents for the treatment of vascular diseases. Chorny et al. reported the use of polylactide nanoparticles incorporating magnetite nanocrystals and encapsulating paclitaxel. *In vitro *studies demonstrated cell growth inhibition with a relatively low dose and brief (five minute) magnetic field exposure. *In vivo *studies performed in a rat carotid artery model of stent restenosis showed a significant benefit over the control group. Additionally, 13.2 ± 2.0 μg of magnetic nanoparticles delivered to the stented carotid segment under a magnetic field were retained in the artery compared to only 3.4 ± 1.9 μg of particles delivered without a magnetic field [80].

Magnetic nanoparticles have also been encapsulated within liposomes. da Silva Gomes et al. synthesized liposomes encapsulating magnetic nanoparticles with an outer polyelectrolyte shell using a layer by layer deposition technique. The lipid vesicles were characterized by dynamic light scattering, cryo-transmission electron microscopy and atomic force microscopy. Polyelectrolyte coated-liposomes were highly stable as they showed no significant membrane disruption or leakage of encapsulated contents in the presence of detergent Triton TX-100 [81].

## Multiple responsive-matrices in drug delivery

Substantial benefits may be gained from the development of polymeric macromolecules that are responsive to small environmental changes and consequently elicit a response. Despite the many advances that have been accomplished, the field of stimuli-responsive biomaterials still faces many challenges. Most of the "smart" materials that have been investigated are primarily focused on a single type of stimulus. Developing a material that is responsive to more than one stimulus may combine the delivery of a drug with other capabilities such as detection, imaging, or feedback. Attention has been focused on materials that respond to more than one stimulus (Table 2).

**Table 2:** Multiple stimuli-sensitive drug delivery.

Stimuli	Carrier type	Payload	Reference
Temperature/pH	PNIPAm/MAA	Vitamin B_12_	[82]
	
	PNIPAm/AA	Isoniazid	[6]
	
	PNIPAm/AAm/VP	Naltrexone	[94]

Light/pH or light/temperature	Polyacrylamide/Salicylideneaniline	None reported	[101]
	
	PSS/PAH/Au	FITC-dextran	[58]

Magnetic/temperature or magnetic/pH	PEO/PPO/PEO/Fe_2_O_3_	Ibuprofen and Eosin Y	[102]
	
	PNIPAm/γ-Fe_2_O_3_/SiO_2_	None reported	[105]
	
	MPEG-b-PDEAEMA-b-PGMA, MPEG-b-PDMAEMA-b-PGMA, PDEAEMA-b-PGMA and MPEG-b-PGMA/Fe_3_O_4 _	Chlorambucil and indomethacin	[109]

### Temperature- and pH-responsive matrices

Temperature- and pH-responsive matrices have been extensively studied in drug delivery because these two parameters often deviate from the norm in diseased tissue. Both environmental changes offer the ability for self-regulated control over the delivery of a drug, avoiding the need for external stimuli. Poly(*N*-isopropylacrylamide-co-methacrylic acid) and PNIPAm are well-established thermo-responsive polymers [6,38,82-94]. These polymers may be combined with pH-responsive polymers, like AA and its alkyl esters such as MAA [6,82,85,94,95]. Zhang et al. prepared temperature and pH-responsive nanoparticles from combinations of PNIPAm and MAA at different molar ratios [82]. The relative permeability of the nanoparticles increased significantly when the temperature was increased from 37°C to 43°C and when the pH decreased from 6 to 4. Nanoparticles encapsulating vitamin B_12 _exhibited a partition coefficient that decreased from 0.8 to 0.3 with increasing temperature and decreased from 0.8 to 0.6 with decreasing pH. Gu et al. prepared PNIPAm-*co*-AA hydrogels with hollow "cages" [6]. They showed that isoniazid (INH), an antitubercular drug, was located inside the cavity of the gel "cages" and also within the shell. The "cages," which were synthesized by SiO_2_-templated polymerization, had a silica core that was subsequently etched away by hydrofluoric acid leaving a hollow interior. The hydrodynamic diameter of the hydrogel "cages" decreased from 367 nm to 200 nm with increasing temperature and decreasing pH. Salehi et al. synthesized an injectable hydrogel system composed of PNIPAm, acrylamide (AAm), and vinyl pyrrolidone (VP) to encapsulate naltrexone, an opiate receptor antagonist [94]. The swelling ratios of the gel increased when the pH decreased from 8.5 to 7.4 and decreased when the temperature increased from 25°C to 37°C. They also performed *in vitro *release studies where a low burst effect and a slow release profile of naltrexone was observed over the course of 28 days.

In addition to PNIPAm, other temperature-responsive polymers have been investigated in dual-responsive drug delivery systems [41,96-100]. Moon et al. prepared and characterized amphiphilic, pH- and temperature-responsive polyaspartamide derivatives, which formed micelles with an average diameter of 100 nm at 25°C [41]. A sol-gel transition was observed when the temperature was increased from 15°C to 25°C and when the pH was increased from 6 to 10. Ding et al. fabricated injectable hydrogels based on glycol chitosan and benzaldehyde-capped poly(ethylene glycol)-*block*-poly(propylene glycol)-*block-*poly(ethylene glycol) (PEO-PPO-PEO) [98]. *In vivo *tests using a rat model demonstrated that the hydrogel underwent a sol-gel transition at physiological conditions. These hydrogels have the ability to encapsulate both hydrophilic and hydrophobic drugs and can control the release profile by varying temperature or pH.

### Light- and pH- or temperature-responsive matrices

Light-responsive materials are combined with a secondary stimulus such as temperature or pH to increase control over drug release. Light responsiveness is usually introduced to a temperature or pH-sensitive material by conjugating a photo-reactive moiety [95]. Jochum et al. synthesized a thermo- and light-responsive polyacrylamide copolymer having salicylideneaniline as a photo-sensitive group [101]. Salicilideneaniline isomerizes from the *enol *to *keto *form and changes its dipole moment upon exposure to UV light. To synthesize the polymer, the authors performed a double polymer analogous reaction of poly(pentafluorophenyl acrylate) (PPFPA) and varied the molar composition of salicylideneaniline from 1 to 15 mol%.

Light- and pH-responsive materials have also been investigated. Angelatos et al. designed light- and pH-responsive polyelectrolyte/gold nanoparticle microcapsules via the layer by layer colloid-templating method [58]. Microcapsules were prepared by depositing the electrolytes poly(sodium 4-styrenesulfonate) (PSS) and poly(allylamine hydrochloride) (PAH) in a layer by layer fashion onto a template of melamine formaldehyde (MF) microparticles. The MF core was subsequently etched away with hydrochloric acid, and gold nanoparticles were introduced into the microcapsule shell. Fluorescein isothiocyanate (FITC)-dextran was encapsulated and was shown to be released after a decrease in pH and upon irradiation of 10 ns pulses of light at 1064 nm.

### Magnetic- and pH- or temperature-responsive matrices

Magnetic fields can be remotely applied to localize drug carriers and to induce a temperature change. There have not been an extensive number of studies focusing on magnetic- and temperature-responsive materials, but this area has received increasing attention within the last few years [102-106]. Luo et al. prepared microspheres by encapsulating silica-coated superparamagnetic magnetite nanoparticle clusters with a crosslinked PNIPAm shell [105]. The microspheres exhibited a temperature-dependent swelling ratio; the hydrodynamic diameter decreased from 750 nm to 500 nm when the temperature increased from 20°C to 60°C. Additionally, the microspheres had greater magnetic responsivity at temperatures higher than the volume phase transition temperature due to the decrease in size at higher temperatures. A faster separation-redispersion behavior of the microspheres was observed at 60°C, above the volume phase transition temperature, compared to that at 25°C. In another study, a different temperature-responsive material, poly(ethyleneimine)-modified poly(ethylene oxide)-poly(propylene oxide)-poly(ethylene oxide) (PEO-PPO-PEO) block copolymer, was used instead of PNIPAm to coat iron oxide nanoparticles [102]. The nanoparticle hydrodynamic diameter decreased from 45 to 25 nm when the temperature increased from 20°C to 35°C. One of the most attractive features of this drug delivery system is the ability to reversibly control the payload release by changing the PEO-PPO-PEO polymer shell conformation. The polymer shell acts as a gate; it is in an extended conformation at room temperature but changes to a coiled conformation upon heating to 37°C. *In vitro *release of hydrophobic and hydrophilic model drugs was achieved by switching the temperature from 37°C to 20°C. In addition, the nanoparticles showed good biocompatibility and effective nerve regeneration when loaded with a ganglioside in a spinal cord injury rat model.

Magnetic- and pH-responsive materials have also been investigated [107-110]. Superparamagnetic Fe_3_O_4 _nanoparticles were coated with different pH-responsive block copolymers [109]. Four different block copolymers, methoxypoly(ethylene glycol)-b- (N,N-diethylamino)ethyl methacrylate-b-poly(glycidyl methacrylate) (MPEG-b-PDEAEMA-b-PGMA), methoxypoly(ethylene glycol)-b-(N,N-diethylamino)methyl methacrylate-b-poly(glycidyl methacrylate) (MPEG-b-PDMAEMA-b-PGMA), PDEAEMA-b-PGMA and MPEG-b-PGMA, were synthesized. The block copolymers were conjugated to the surface of Fe_3_O_4 _nanoparticles stabilized with HClO_4 _via a ligand exchange method. The authors performed drug release studies with MPEG-b-PDEAEMA-b-PGMA-Fe_3_O_4 _and MPEG-b-PDMAEMA-b-PGMA-Fe_3_O_4 _nanocarriers encapsulating chlorambucil (CLB), an anti-cancer agent, and indomethacin (IND), an anti-inflammatory agent. Their results showed that upon a decrease in pH (below the *p*K_a _of each drug), the percentage of drug release increased up to 90% for CLB and 70% for IND. At pH 7.4 the percent of drug release for both IND and CLB was approximately 25%.

## Concluding remarks

The ability to alter the biodistribution of a drug by modulating its release profile through the use of smart polymers could transform drug delivery from passive controlled release to active stimuli-regulated delivery. Altering the drug biodistribution has the ability to reduce toxicity and side effects while improving therapeutic outcomes due to the ability to deliver higher doses of drug to the site of interest. The stimuli-responsive polymers reviewed here serve to provide a snapshot of the utility and complexity of polymers that can sense, process, and respond to stimuli in modulating the release of a drug. Stimuli-responsive drug delivery vehicles come in the form of polymersomes [111,112], liposomes [113-115], micelles [116-118], and dendrimers [119,120]. All of these systems aim to deliver an effective dose of drug at a specific time and place.

Despite many advances, there are still numerous challenges and opportunities that exist to translate responsive polymers from the laboratory the clinic. There is a need to develop polymers with greater sensitivity to a more diverse range of stimuli. In terms of biochemical signals or biomarkers, this is usually in the range of nano to picomolar concentrations [121]. This may require both a highly sensitive sensing mechanism and/or an amplification system to elicit a response from the polymer. In terms of the physical microenvironment, there are only minor differences in pH and temperature between diseased and normal tissues. Therefore, smart polymers must be able to accurately sense the changes in their surroundings to target drug release.

There is also a significant opportunity for smart polymers to respond to multiple stimuli. Hybrid polymers created in this manner will offer more parameters to tune drug delivery, which may be necessary for more complex and dynamic environments. It is worth noting that in addition to drug delivery applications, smart polymers in general have broad applications in tissue engineering and regenerative medicine (e.g. as injectable systems for delivery of cells or self-regulating scaffolds for cell growth or infiltration), and in actuators (e.g. as smart valves and coating in microfluidics or shape memory devices). Given the continuous development of new responsive polymer compositions, we expect increasingly elaborate and versatile drug carriers to be introduced in the future.

## Competing interests

The authors declare that they have no competing interests.

## Authors' contributions

JY performed the experiments of transmission electron microscope (TEM), paclitaxel release study, and drafted the manuscript. JY, DA, and GY wrote the manuscript. DTA is responsible for the overall content. All authors read and approved the final manuscript.
